# The requirement of the mitochondrial protein NDUFS8 for angiogenesis

**DOI:** 10.1038/s41419-024-06636-3

**Published:** 2024-04-09

**Authors:** Qian-wei Xiong, Kun Jiang, Xiao-wei Shen, Zhou-rui Ma, Xiang-ming Yan, Hao Xia, Xu Cao

**Affiliations:** 1 Department of Urology Surgery, Children’s Hospital of Soochow University, Suzhou, China; 2 Vascular Surgery Department, Kunshan Traditional Chinese Medicine Hospital, Kunshan, China; 3 Department of General Surgery, QingPu Branch of Zhongshan Hospital Affiliated to Fudan University, QingPu District Central Hospital Shanghai, Shanghai, China; 4 Department of Burns and Plastic Surgery, Children’s Hospital of Soochow University, Suzhou, China; 5 Department of Pediatric Emergency and Critical Care Medicine, Xin Hua Hospital, Shanghai Jiao Tong University School of Medicine, Shanghai, China

**Keywords:** Angiogenesis, Stress signalling

## Abstract

Mitochondria are important for the activation of endothelial cells and the process of angiogenesis. NDUFS8 (NADH:ubiquinone oxidoreductase core subunit S8) is a protein that plays a critical role in the function of mitochondrial Complex I. We aimed to investigate the potential involvement of NDUFS8 in angiogenesis. In human umbilical vein endothelial cells (HUVECs) and other endothelial cell types, we employed viral shRNA to silence NDUFS8 or employed the CRISPR/Cas9 method to knockout (KO) it, resulting in impaired mitochondrial functions in the endothelial cells, causing reduction in mitochondrial oxygen consumption and Complex I activity, decreased ATP production, mitochondrial depolarization, increased oxidative stress and reactive oxygen species (ROS) production, and enhanced lipid oxidation. Significantly, NDUFS8 silencing or KO hindered cell proliferation, migration, and capillary tube formation in cultured endothelial cells. In addition, there was a moderate increase in apoptosis within NDUFS8-depleted endothelial cells. Conversely, ectopic overexpression of NDUFS8 demonstrated a pro-angiogenic impact, enhancing cell proliferation, migration, and capillary tube formation in HUVECs and other endothelial cells. NDUFS8 is pivotal for Akt-mTOR cascade activation in endothelial cells. Depleting NDUFS8 inhibited Akt-mTOR activation, reversible with exogenous ATP in HUVECs. Conversely, NDUFS8 overexpression boosted Akt-mTOR activation. Furthermore, the inhibitory effects of NDUFS8 knockdown on cell proliferation, migration, and capillary tube formation were rescued by Akt re-activation via a constitutively-active Akt1. In vivo experiments using an endothelial-specific NDUFS8 shRNA adeno-associated virus (AAV), administered via intravitreous injection, revealed that endothelial knockdown of NDUFS8 inhibited retinal angiogenesis. ATP reduction, oxidative stress, and enhanced lipid oxidation were detected in mouse retinal tissues with endothelial knockdown of NDUFS8. Lastly, we observed an increase in NDUFS8 expression in retinal proliferative membrane tissues obtained from human patients with proliferative diabetic retinopathy. Our findings underscore the essential role of the mitochondrial protein NDUFS8 in regulating endothelial cell activation and angiogenesis.

## Introduction

Angiogenesis is the process by which new blood vessels form from pre-existing ones, and it is a fundamental biological mechanism with critical implications for various physiological and pathological conditions [1–5]. Central to angiogenesis are endothelial cells, which line the inner surface of blood vessels [6–8]. When angiogenesis is triggered, typically in response to signals like tissue injury, low oxygen levels and release of growth factors, endothelial cells become activated [1–5]. Endothelial cells then undergo changes including increased permeability and adhesion molecule expression [1–5]. These endothelial cells then migrate to the target site, secrete enzymes to break down the surrounding tissue, proliferate, and eventually organize into tubular sprouts, forming new blood vessels [1–5]. Dysregulation of angiogenesis can lead to various diseases, including cancer, diabetes, and cardiovascular diseases, it is therefore extremely important to understand the mechanism of endothelial cell activation and angiogenesis [1–5].

Endothelial cells require energy in the form of ATP to undergo activation, and mitochondria serve as the primary source of this energy [9, 10]. The energy is crucial for processes such as sprouting, migration, and proliferation of endothelial cells, allowing them to form new blood vessels efficiently [11, 12]. Without functional mitochondria, endothelial cells will lack the energy needed for this activation, compromising their ability to respond to physiological and pathological stimuli effectively [11, 12]. Apart from their role in energy production, mitochondria also actively engage in the regulation of several critical cellular processes [13–18]. These include cellular differentiation, signal transduction, apoptosis, as well as the control of cell growth and the cell cycle in endothelial cells [13–18]. Thus, proper mitochondrial function is vital for endothelial cell activation and angiogenesis process.

Mitochondrial Complex I, also known as NADH:ubiquinone oxidoreductase or respiratory Complex I, is a crucial component of the mitochondrial electron transport chain (ETC) [19, 20]. NDUFS8 (NADH:ubiquinone oxidoreductase core subunit S8) is an important component of the complex [21, 22]. NDUFS8’s role within Complex I involves facilitating the transfer of electrons from NADH to ubiquinone, a pivotal step in oxidative phosphorylation (OXPHOS) and energy production [21, 22]. This electron transfer leads to the pumping of protons across the inner mitochondrial membrane, creating a proton gradient essential for ATP synthesis [21, 22]. The current study explored the potential functional significance and underlying mechanism of NDUFS8 in endothelial cell activation and angiogenesis.

## Materials and methods

### Reagents

Chemicals were sourced from Sigma-Aldrich (St. Louis, MO) unless specified otherwise. The anti-NDUFS8 antibody was obtained from Abcam (Cambridge, UK). The anti-NDUFS1 antibody was provided by Cell Signaling Tech (Danvers, MA). All other antibodies were described in our previous studies [23, 24]. Fluorescence dyes were obtained from Thermo-Fisher Invitrogen (Soochow, China), and all viral constructs were supplied by Genechem (Shanghai, China).

### Cells

Human umbilical vein endothelial cells (HUVECs), human microvascular endothelial cells (hRMEC), human dermal endothelial cells (hDEC), and human cerebral microvascular endothelial cells (hCMEC) were reported in our previous studies [23, 24]. These endothelial cells were always maintained under pro-angiogenic active conditions [25], and their genotypes were confirmed through short tandem repeat (STR) analysis, population doubling time measurement, and morphological examination.

### NDUFS8 short hairpin RNA (shRNA)

NDUFS8 silencing was achieved by using lentivirus-packaged NDUFS8 shRNAs, kdNDUFS8-sh2, and kdNDUFS8-sh5, targeting different sequences (Genechem, Shanghai, China). Endothelial cells were infected with the virus (multiplicity of infection/MOI = 15) for 48 h and selected with puromycin for 6–7 passages. Control endothelial cells were transduced with scramble control non-sense shRNA (“kdC” [24]). In the resulting stable cells, NDUFS8 expression was verified at both mRNA and protein levels. For NDUFS8 silencing in vivo, the NDUFS8 shRNA sequence (mouse, Genechem) was sub-cloned into the AAV5-TIE1 construct (reported previously [23, 25, 26]) to generate AAV.

### NDUFS8 knockout (KO)

NDUFS8 KO was accomplished in stable HUVECs with the clustered regularly interspaced short palindromic repeats (CRISPR)-associated protein 9 (Cas9)-expressing construct (described in our previous studies [23, 24]). Lentivirus-packaged CRISPR/Cas9-NDUFS8-KO construct [24] (with sgRNA against human *NDUFS8*, Genechem) was used for infection, followed by puromycin selection for 4–5 passages. NDUFS8 KO was confirmed through PCR assays, establishing single stable NDUFS8 KO HUVECs (“koNDUFS8”). Control HUVECs were stably transduced with lentiviral CRISPR/Cas9-control construct with scramble control non-sense sgRNA (“sgC”).

### Ectopic overexpression of NDUFS8

NDUFS8 overexpression involved infecting HUVECs or other endothelial cells with lentivirus encoding NDUFS8-overexpressing (hNDUFS8[NM_002496.4], 6532 bp) construct (GV248 vector, no EGFP tag, Genechem) at MOI = 15. After puromycin selection for 5–6 passages, stable cells, “oeNDUFS8,” were established. Control endothelial cells were transduced with the lentiviral empty vector (“Vec“ [23, 24]). NDUFS8 expression was confirmed at both mRNA and protein levels.

### Other assays

Various in vitro cellular functional assays, gene/protein detections, and biochemical assays were performed as previously described in detail [23, 24], including assessments of cell proliferation (EdU-nuclear staining), migration (“Transwell” assay), invasion (“Matrigel Transwell” assay), capillary tube formation, reactive oxygen species (ROS) production (through MitoSOX staining), mitochondrial depolarization (JC-1 staining), Caspase-3 and Caspase-9 activity, TUNEL-nuclear staining, mitochondrial Complex I activity, and cellular ATP levels. Quantitative real-time PCR (qRT-PCR) and Western blotting followed established protocols [25, 27–31]. For cell migration/invasion assay, cells were stained with crystal violet. The cytochrome C ELISA assays have been described in detail elsewhere [32, 33]. Figure S2 listed the uncropped blotting images.

### Animal studies

The adult C57BL/6 mice and AAV intravitreous injection procedures were described early in our previous studies [23, 24]. Retinal vasculature was stained with isolectin B4 (IB4) as previously detailed [23, 24, 26, 34]. The protocols were approved by the Institutional Animal Care and Use Committee and the Ethic Committee of Soochow University, following ARVO (Association for Research in Vision and Ophthalmology) statement guidelines.

### Measuring the ratio of reduced glutathione (GSH) to oxidized glutathione (GSSG)

A GSH/GSSG ratio kit was procured from Thermo Fisher Scientific (Suzhou, China). The lysates of murine retinal tissues were mixed with 5,5’-Dithio-bis(2-nitrobenzoic acid) (DTNB), glutathione reductase, and NADPH. Subsequently, the lysates were further mixed with a reaction solution, and the absorbance at 430 nm was recorded over a period of five minutes using a spectrophotometer. A standard curve was established with GSH and GSSG standards to determine their concentrations in the lysates, and the ratio was normalized to the protein concentration.

### Thiobarbituric acid reactive substances (TBAR) assay

A TBAR assay kit was from Thermo Fisher Scientific (Suzhou, China). Tissue protein lysates were allowed to react with thiobarbituric acid (TBA) to form the TBAR complex. After cooling and centrifugation to eliminate any precipitate, the absorbance at 545 nm was measured using a spectrophotometer.

### Mitochondrial oxygen consumption

Mitochondrial oxygen consumption was monitored via MitoXpress Xtra dye (Cayman Chemical Company, Shanghai, China) according to the manufacturer’s protocols. MitoXpress Xtra is quenched by O_2_, and thus the amount of fluorescence signal is inversely proportional to the amount of extracellular O_2_. Briefly, HUVECs were grown in 96-well plates and were labeled with MitoXpress Xtra. The wells were sealed with 100 µL 19HS mineral oil (Cayman Chemical Company). The plate was then measured at 20 min intervals for a total of 100 min to ensure that the fluorescent signal was stable. Time-resolved fluorescence measurements were performed at 380 nm excitation and 650 nm emission with a dual delay of 30 μs and 70 μs using a fluorescence microplate reader (Corning, NY). The fluorescence intensity optical density was recorded.

### Akt1 mutation

The lentiviral particles containing the constitutively active S473D mutant Akt1 (caAkt1) were provided by Dr. Chen [35], which were added into cultured HUVECs. The establishment of stable cells expressing caAkt1 was achieved through puromycin-based selection.

### Analyzing human tissues

Human tissues used in this study were obtained from patients who provided written informed consent and were part of Dr. Jiang’s group [25, 28]. The use of these tissues was previously reported in published studies [25, 28], and all research protocols involving human samples were ethically approved by the Ethics Committee of Soochow University.

### Statistical analyses

Statistical analyses were conducted using normally-distributed data, expressed as means ± standard deviation (SD). To assess significance, one-way ANOVA followed by Scheffe’s f-test (for comparisons involving three or more groups, using SPSS 23.0) or the two-tailed unpaired t-test (for comparisons between two groups, using Excel 2007) were employed. Statistical significance was defined as *P*-values less than 0.05.

## Results

### NDUFS8 silencing impairs mitochondrial functions in cultured endothelial cells

**Fig. 1:**
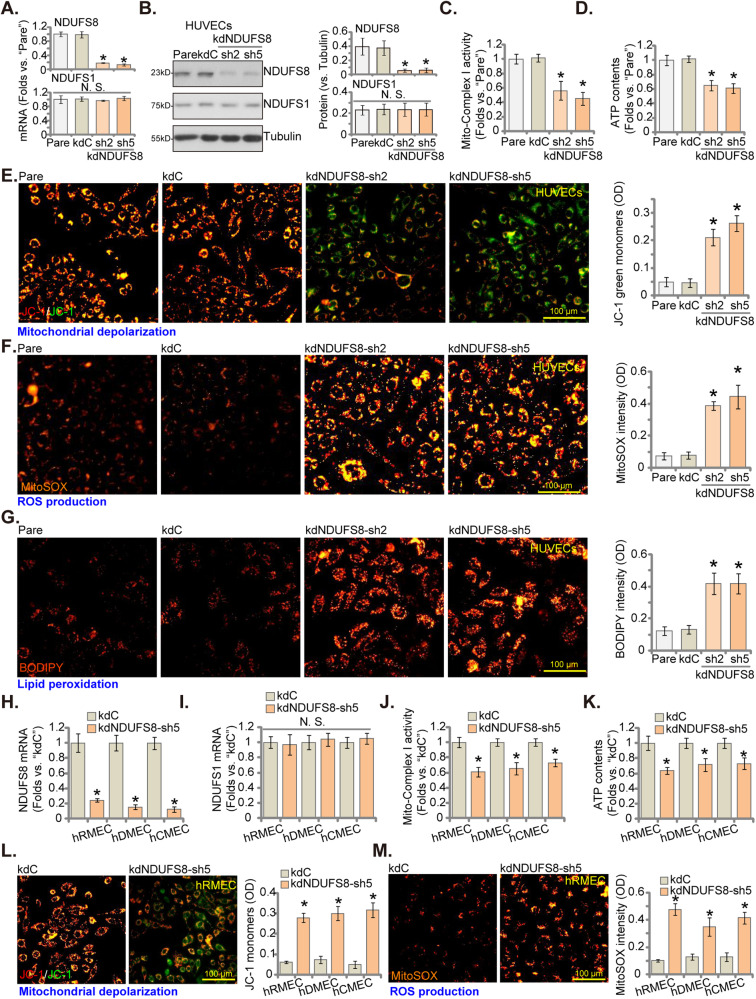
NDUFS8 silencing impairs mitochondrial functions in cultured endothelial cells. NDUFS1/NDUFS8 expression in stable HUVECs treated with NDUFS8 shRNA (“kdNDUFS8-sh2” and “kdNDUFS8-sh5”) or the scramble non-sense shRNA (“kdC”) was shown (**A**, **B**). Following a 24 h culture, the mitochondrial respiratory chain Complex I activity (**C**), cellular ATP levels (**D**), reduction in mitochondrial membrane potential (tested via mitochondrial JC-1 staining, **E**), ROS contents (measured using MitoSOX dye, **F**), and lipid peroxidation (via BODIPY staining, **G**) were shown. Similarly, human microvascular endothelial cells (hRMEC), human dermal endothelial cells (hDEC), and human cerebral microvascular endothelial cells (hCMEC) with either kdNDUFS8-sh5 or kdC were established. *NDUFS8* (**H**) and *NDUFS1* (**I**) mRNA expression was quantified, followed by a 24 h culture and examination of mitochondrial respiratory chain Complex I activity (**J**), cellular ATP content (**K**), mitochondrial depolarization (by measuring JC-1 green monomers intensity, **L**), and ROS production (by measuring MitoSOX intensity, **M**). “Pare” denotes the parental control endothelial cells. The data are presented as mean ± standard deviation (SD, *n* = 5). *P < 0.05 compared to “Pare”/“kdC” cells. “N. S.” represents non-statistically significant disparities (*P* > 0.05). These experiments were repeated five times, yielding consistent results. Scale bar = 100 μm.

To investigate the potential function of NDUFS8 in endothelial cells, we employed the shRNA strategy to silence NDUFS8. Initially, six distinct lentivirus-packed shRNAs were individually transduced into primary cultured HUVECs [23, 24], followed by the establishment of stable cells through puromycin-based selection. Among the tested shRNAs, only two, namely kdNDUFS8-sh2 and kdNDUFS8-sh5, achieved substantial downregulation of *NDUFS8* mRNA and protein levels in HUVECs, as illustrated in Fig. 1A, B. Importantly, the expression of the control gene *NDUFS1* remained unchanged in HUVECs subjected to NDUFS8 shRNA treatment (Fig. 1A, B). As expected, the control shRNA, referred as “kdC,” had no significant impact on the expression of NDUFS1 and NDUFS8 in HUVECs (Fig. 1A, B).

The silencing of NDUFS8 through targeted shRNAs led to the disruption of mitochondrial functions in HUVECs. MitoXpress Xtra is quenched by O_2_ and thus the amount of fluorescence intensity is inversely proportional to the amount of O_2_. As shown, the mitochondrial oxygen consumption, reflected by the increased MitoXpress Xtra fluorescence intensity over time, was significantly decreased in NDUFS8 shRNA-expressing HUVECs (Figure S1A). Moreover, mitochondrial Complex I activity (Fig. 1C) and cellular ATP levels (Fig. 1D) were significantly reduced in HUVECs expressing kdNDUFS8-sh2 or kdNDUFS8-sh5. Moreover, this silencing resulted in mitochondrial depolarization, evident from the conversion of JC-1 red aggregates to green monomers (Fig. 1E). Concomitantly, there was a marked increase in oxidative injury and the production of ROS in NDUFS8-silenced HUVECs, as demonstrated by the elevated MitoSOX red fluorescence intensity (Fig. 1F). In addition, enhanced lipid peroxidation was observed in NDUFS8-silenced HUVECs, as indicated by increased BODIPY fluorescence intensity (Fig. 1G). In contrast, treatment with the control shRNA, kdC, had no significant impact on mitochondrial functions in HUVECs (Fig. 1C–G).

Furthermore, the lentivirus-packed kdNDUFS8-sh5 was introduced into various other endothelial cell types, including human microvascular endothelial cells (hRMEC), human dermal endothelial cells (hDEC), and human cerebral microvascular endothelial cells (hCMEC). kdNDUFS8-sh5 led to a substantial downregulation of *NDUFS8* mRNA (Fig. 1H), while *NDUFS1* mRNA expression remained unaffected (Fig. 1I). In these endothelial cells, silencing of NDUFS8 using kdNDUFS8-sh5 similarly inhibited mitochondrial Complex I activity (Fig. 1J), reduced cellular ATP content (Fig. 1K), induced mitochondrial depolarization (Fig. 1L), and triggered ROS production (Fig. 1M). These results support that NDUFS8 is important for maintaining mitochondrial functions in various endothelial cells.

### NDUFS8 silencing impedes in vitro angiogenesis in cultured endothelial cells

**Fig. 2:**
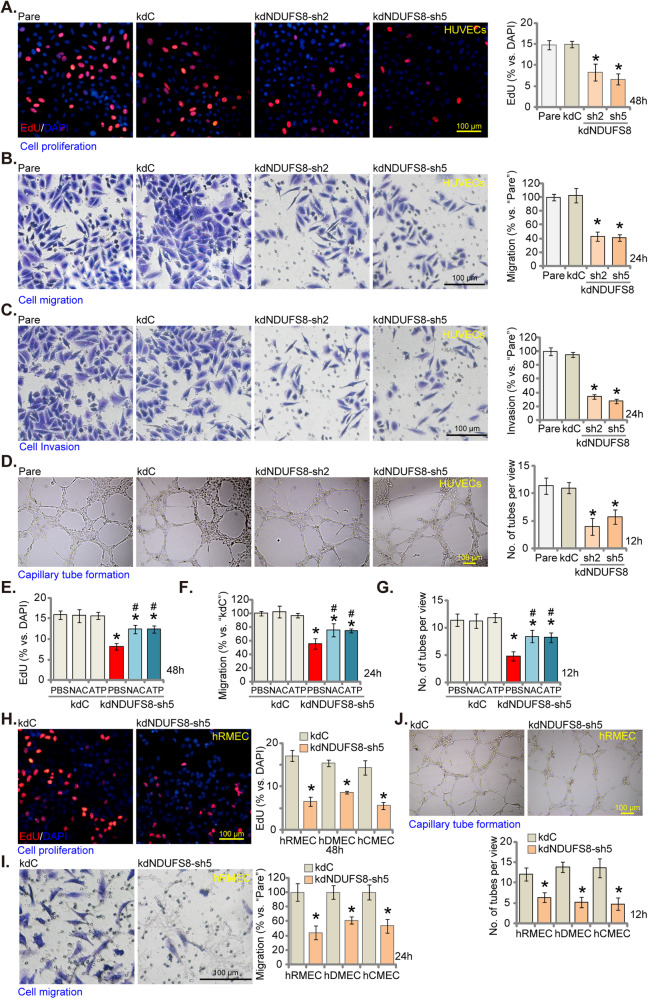
NDUFS8 silencing impedes in vitro angiogenesis in cultured endothelial cells. HUVECs treated with NDUFS8 shRNA (“kdNDUFS8-sh2” and “kdNDUFS8-sh5”) or the scramble non-sense shRNA (“kdC”) were cultivated for designated hours, cell proliferation (EdU incorporation in nuclei, **A**), in vitro cell migration (**B**) and invasion (**C**) as well as capillary tube formation (**D**) were examined. HUVECs with “kdNDUFS8-sh5” or “kdC” were treated with antioxidant N-Acetylcysteine (NAC, 500 μM) or ATP (1 mM) for designated hours, cell proliferation (EdU incorporation in nuclei, **E**), in vitro cell migration (**F**) and capillary tube formation (**G**) were examined, with results quantified. Human microvascular endothelial cells (hRMEC), human dermal endothelial cells (hDEC), and human cerebral microvascular endothelial cells (hCMEC) with either kdNDUFS8-sh5 or kdC were cultured for designated hours, cell proliferation (**H**), in vitro cell migration (**I**), and capillary tube formation (**J**) were examined similarly. “Pare” denotes the parental control endothelial cells. The data are presented as mean ± standard deviation (SD, *n* = 5). *P < 0.05 compared to “Pare”/“kdC” cells. ^#^*P* < 0.05 compared to “PBS” pretreatment (**E**–**G**). These experiments were repeated five times, yielding consistent results. Scale bar = 100 μm.

We next explored the effect of NDUFS8 silencing on in vitro angiogenesis activity in endothelial cells. NDUFS8 silencing by kdNDUFS8-sh2 and kdNDUFS8-sh5 (see Fig. 1) inhibited proliferation of HUVECs and significantly decreased the proportion of nuclei displaying positive EdU staining (Fig. 2A). Moreover, NDUFS8 silencing impaired in vitro cell migration and invasion of HUVECs, as assessed through “Transwell” (Fig. 2B) and “Matrigel Transwell” (Fig. 2C) assays, respectively. In addition, capillary tube formation was impeded in HUVECs following NDUFS8 silencing (Fig. 2D). Treatment with the control shRNA, kdC, had no discernible impact on proliferation (Fig. 2A), migration (Fig. 2B), invasion (Fig. 2C), or capillary tube formation (Fig. 2D) in HUVECs. Disruption of mitochondrial functions is likely the key factor behind the inhibition of angiogenesis ability observed in endothelial cells where NDUFS8 was silenced. The potent antioxidant N-acetylcysteine (NAC) and ATP supplement significantly alleviated kdNDUFS8-sh5-induced inhibitory effects on cell proliferation (Fig. 2E), migration (Fig. 2F) and capillary tube formation (Fig. 2G) in HUVECs.

In other endothelial cell types, including hRMEC, hDEC, and hCMEC, NDUFS8 silencing with kdNDUFS8-sh5 (see Fig. 1) similarly impeded in vitro angiogenesis. Specifically, kdNDUFS8-sh5 inhibited proliferation (EdU incorporation, Fig. 2H), migration (Fig. 2I), and capillary tube formation (Fig. 2J) in the endothelial cells. These results support that NDUFS8 is important for in vitro angiogenesis of endothelial cells.

### Induction of apoptosis by NDUFS8 silencing in cultured endothelial cells

**Fig. 3:**
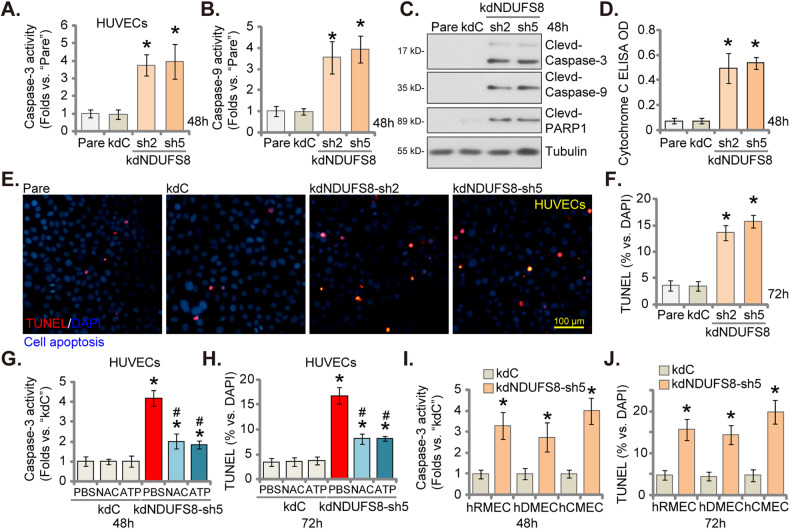
Induction of apoptosis by NDUFS8 silencing in cultured endothelial cells. HUVECs treated with NDUFS8 shRNA (“kdNDUFS8-sh2” and “kdNDUFS8-sh5”) or the scramble non-sense shRNA (“kdC”) were cultivated for designated hours, Caspase-3 (**A**) and Caspase-9 (**B**) activities were measured; Expression of listed apoptosis proteins was shown (**C**); Cytosol Cytochrome C release was measured via an ELISA kit, with its intensity recorded (**D**); Cell apoptosis was measured via nuclear TUNEL staining (**E**, **F**) assay. HUVECs with “kdNDUFS8-sh5” or “kdC” were treated with antioxidant N-Acetylcysteine (NAC, 500 μM) or ATP (1 mM) for designated hours, the Caspase-3 (**G**) and apoptosis (via measuring TUNEL-positive nuclei ratio, **H**) were examined, with results quantified. Human microvascular endothelial cells (hRMEC), human dermal endothelial cells (hDEC), and human cerebral microvascular endothelial cells (hCMEC) with either kdNDUFS8-sh5 or kdC were cultured for designated hours, Caspase-3 activity (**I**) and cell apoptosis (**J**) were measured similarly, with results quantified. “Pare” denotes the parental control endothelial cells. The data are presented as mean ± standard deviation (SD, *n* = 5). *P < 0.05 compared to “Pare”/“kdC” cells. ^#^*P* < 0.05 compared to “PBS” pretreatment (**G**, **H**). These experiments were repeated five times, yielding consistent results. Scale bar = 100 μm.

We investigated whether NDUFS8 silencing could induce apoptosis in endothelial cells. NDUFS8 silencing using kdNDUFS8-sh2 and kdNDUFS8-sh5 (see Figs. 1 and 2) increased Caspase-3 activity (Fig. 3A) and Caspase-9 activity (Fig. 3B) in primary cultured HUVECs. Furthermore, levels of cleaved-Caspase-3, cleaved-Caspase-9, and cleaved-Poly (ADP-ribose) polymerase (PARP1) were elevated in NDUFS8-silenced HUVECs (Fig. 3C). Moreover, increased Cytochrome C release into the cytosol of HUVECs was confirmed via ELISA assay (Fig. 3D), and it is a crucial step for mitochondrial apoptosis cascade activation [36–39]. Moderate but significant apoptosis was observed in HUVECs expressing kdNDUFS8, as indicated by an increased ratio of TUNEL-positive nuclei (Fig. 3E, F). Notably, treatment with the control shRNA, kdC, did not activate Caspases and apoptosis in HUVECs (Fig. 3A–F).

Importantly, NAC and ATP largely inhibited kdNDUFS8-sh5-induced Caspase-3 activation (Fig. 3G) and apoptosis (via measuring TUNEL-nuclei ratio, Fig. 3H) in HUVECs, suggesting that mitochondrial impairment is a key reason of NDUFS8 silencing-induced apoptosis in endothelial cells. In hRMEC, hDEC, and hCMEC, NDUFS8 silencing with kdNDUFS8-sh5 (see Fig. 1) also induced apoptosis activation, as evidenced by increased Caspase-3 activity (Fig. 3I) and an elevated ratio of TUNEL-positive nuclei (Fig. 3J).

### NDUFS8 knockout impairs mitochondrial functions and impeded in vitro angiogenesis in cultured endothelial cells

**Fig. 4:**
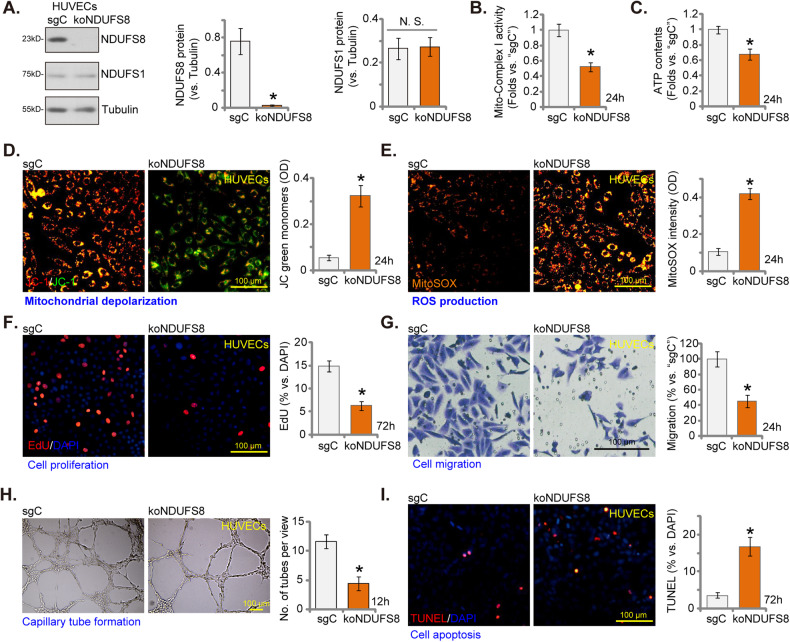
NDUFS8 knockout impairs mitochondrial functions and impeded in vitro angiogenesis in cultured endothelial cells. The protein expression of NDUFS1/NDUFS8 in stable HUVECs with the Cas9-expressing construct plus the CRISPR/Cas9-NDUFS8-KO construct (“koNDUFS8”) or the control construct (“sgC”) was shown (**A**); Following culture of designated hours, the mitochondrial respiratory chain Complex I activity (**B**), cellular ATP levels (**C**), reduction in mitochondrial membrane potential (measured via mitochondrial JC-1 staining, **D**), ROS levels (measured using MitoSOX dye, **E**) were tested; Cell proliferation (measured via quantifying nuclear EdU incorporation, **F**), in vitro cell migration (**G**) and capillary tube formation (**H**) were also examined; Cell apoptosis was measured via quantifying nuclear TUNEL ratio (**I**) were tested as well. The data are presented as mean ± standard deviation (SD, *n* = 5). *P < 0.05 compared to “sgC” cells. “N. S.” represents non-statistically significant disparities (*P* > 0.05). These experiments were repeated five times, yielding consistent results. Scale bar = 100 μm.

To further substantiate the role of NDUFS8 in endothelial cell activation and angiogenesis, we employed the CRISPR/Cas9 strategy to knockout (KO) NDUFS8. Specifically, a lentiviral CRISPR/Cas9-NDUFS8-KO construct with sgRNA targeting *NDUFS8* was stably transduced into Cas9-expressing HUVECs. After puromycin selection and verification of NDUFS8 KO, single stable NDUFS8 KO HUVECs (“koNDUFS8”) were established. In comparison to control cells harboring the lentiviral CRISPR/Cas9-KO construct with non-sense control sgRNA (“sgC”), koNDUFS8 HUVECs exhibited substantial reduction in NDUFS8 protein expression (Fig. 4A), while the control NDUFS1 protein expression remained unaltered (Fig. 4A).

CRISPR/Cas9-induced NDUFS8 KO impaired mitochondrial functions, resulting in decreased mitochondrial Complex I activity (Fig. 4B) and cellular ATP contents (Fig. 4C) in HUVECs. Mitochondrial depolarization, as indicated by JC-1 green monomers’ accumulation, was also observed (Fig. 4D). MitoXpress Xtra assay results demonstrated that NDUFS8 KO potently inhibited the mitochondrial oxygen consumption in HUVECs (Figure S1B). Moreover, koNDUFS8 HUVECs displayed significant ROS production and oxidative injury, as evidenced by the increase in MitoSOX red fluorescence intensity (Fig. 4E).

NDUFS8 KO in HUVECs led to a reduction in in vitro angiogenesis ability. The proportion of EdU-positive nuclei was substantially decreased in koNDUFS8 HUVECs (Fig. 4F), indicating impaired proliferation. In addition, NDUFS8 KO inhibited in vitro migration (Fig. 4G) of HUVECs. Furthermore, capillary tube formation was suppressed after NDUFS8 KO, resulting in a decreased number of formed capillary tubes in koNDUFS8 HUVECs (Fig. 4H). Contrarily, apoptosis was detected in koNDUFS8 HUVECs, supported by the increased TUNEL-positive nuclei ratio (Fig. 4I). These findings provided strong support for the notion that NDUFS8 KO impairs mitochondrial function and impedes in vitro angiogenesis in cultured endothelial cells.

### Pro-angiogenic effect of NDUFS8 overexpression in endothelial cells

**Fig. 5:**
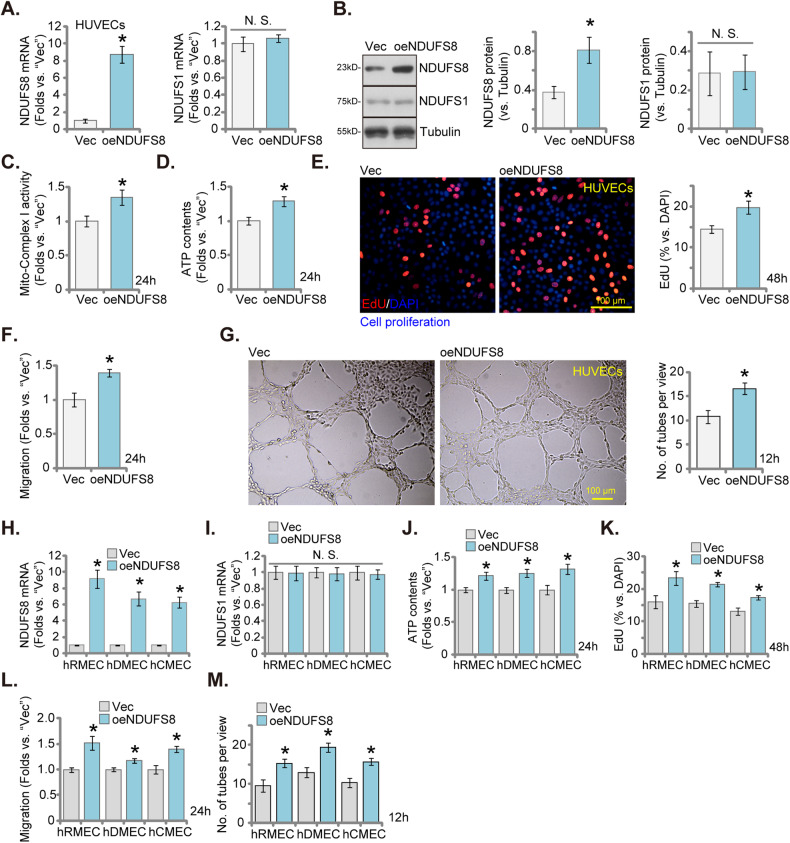
Pro-angiogenic effect of NDUFS8 overexpression in endothelial cells. NDUFS1/NDUFS8 expression in designated endothelial cells (HUVECs, hRMEC, hDEC, and hCMEC) treated with the lentivirus-packed NDUFS8-overexpressing construct (“oeNDUFS8”) or vector control (“Vec”) was shown (**A**, **B**, **H**, **I**). Following culture of designated hours, the mitochondrial respiratory chain Complex I activity (**C**) and cellular ATP levels (**D**, **J**) were measured. Cell proliferation (measured via quantifying nuclear EdU incorporation, **E**, **K**), in vitro cell migration (**F**, **L**) as well as capillary tube formation (**G**, **M**) were also examined, with results quantified. The data are presented as mean ± standard deviation (SD, *n* = 5). *P < 0.05 compared to “Vec” cells. “N. S.” represents non-statistically significant disparities (*P* > 0.05). These experiments were repeated five times, yielding consistent results. Scale bar = 100 μm.

We hypothesized that increasing NDUFS8 expression shall then promote angiogenesis in endothelial cells. To test this hypothesis, HUVECs were transduced with a lentivirus-packed NDUFS8-overexpressing construct and stable cells were established through puromycin-based selection. These cells were designated as “oeNDUFS8” HUVECs. In comparison to vector control cells (“Vec”), oeNDUFS8 HUVECs exhibited a significant increase in *NDUFS8* mRNA (Fig. 5A) and protein (Fig. 5B) expression levels, while *NDUFS1* mRNA (Fig. 5A) and protein (Fig. 5B) expression was unchanged. Importantly, in oeNDUFS8 HUVECs, mitochondrial Complex I activity (Fig. 5C) and the cellular ATP content (Fig. 5D) were augmented. The ectopic overexpression of NDUFS8 had a pro-angiogenic effect, promoting cell proliferation (EdU incorporation) in HUVECs (Fig. 5E). In addition, in vitro cell migration (Fig. 5F) was accelerated in oeNDUFS8 HUVECs. Furthermore, NDUFS8 overexpression facilitated capillary tube formation in HUVECs (Fig. 5G), underscoring its pro-angiogenic activity.

The same lentiviral construct was employed to induce stable NDUFS8 overexpression (“oeNDUFS8”) in other endothelial cells, including hRMEC, hDEC, and hCMEC. This resulted in robust upregulation of NDUFS8 *mRNA* in the endothelial cells (Fig. 5H), while *NDUFS1* mRNA expression remained unaltered (Fig. 5I). In the oeNDUFS8 endothelial cells, cellular ATP contents were augmented (Fig. 5J). Importantly, NDUFS8 overexpression also induced pro-angiogenic activity in these endothelial cells, enhancing cell proliferation (Fig. 5K), in vitro cell migration (Fig. 5L), and capillary tube formation (Fig. 5M).

### NDUFS8 plays a crucial role in promoting Akt-mTOR pathway activation in endothelial cells

**Fig. 6:**
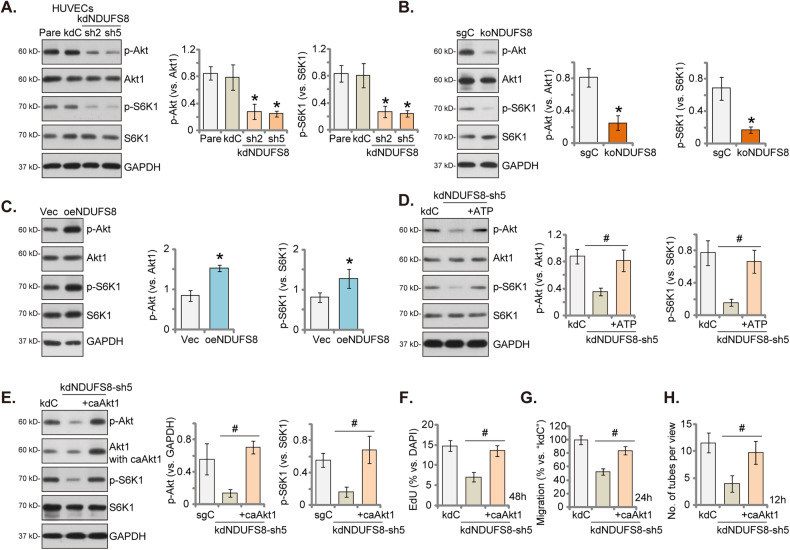
NDUFS8 plays a crucial role in promoting Akt-mTOR pathway activation in endothelial cells. Expression of listed proteins in HUVECs with NDUFS8 shRNA (“kdNDUFS8-sh2” and “kdNDUFS8-sh5”), the scramble non-sense shRNA (“kdC”) (**A**), the Cas9-expressing construct plus the CRISPR/Cas9-NDUFS8-KO construct (“koNDUFS8”), the control construct (“sgC”) (**B**), the lentivirus-packed NDUFS8-overexpressing construct (“oeNDUFS8”) or vector control (“Vec”) (**C**) was shown. HUVECs with “kdNDUFS8-sh5” or “kdC” were treated with ATP (1 mM) for 12 h, expression of listed proteins was shown (**D**). The kdNDUFS8-sh5-expressing HUVECs were further stably transduced with or without constitutively-active (S473D) mutant Akt1 (caAkt1), expression of listed proteins was shown (**E**); Cells were further cultivated for indicated hours, cell proliferation (EdU incorporation in nuclei, **F**), in vitro cell migration (**G**) and capillary tube formation (**H**) were examined, with results quantified. The data are presented as mean ± standard deviation (SD, *n* = 5). *P < 0.05 compared to “kdC”/“sgC”/“Vec” cells. ^#^*P* < 0.05 (**D**–**H**). These experiments were repeated five times, yielding consistent results.

ATP stands at the core of Akt activation, functioning not only as the primary energy source driving phosphorylation reactions but also as the critical supplier of phosphate groups during kinase-mediated phosphorylation events [40–44]. Its dual role underscores the indispensable role of ATP in orchestrating the intricate processes that culminate in Akt activation [40–44]. Given that NDUFS8 plays a pivotal role in ATP production and that Akt-mTOR activation is crucial for angiogenesis [45–47], we conducted experiments to investigate whether NDUFS8 is important for Akt-mTOR activation in endothelial cells. In HUVECs, the silencing of NDUFS8 through shRNA, specifically with kdNDUFS8-sh2 or kdNDUFS8 -sh5 (see Figs. 1 and 2), resulted in a significant reduction in the phosphorylation of Akt and p70S6 kinase 1 (S6K1) (Fig. 6A). There was no observable change in the total protein expression of Akt1 and S6K1 (Fig. 6A). Furthermore, CRISPR/Cas9-mediated KO of NDUFS8 (see Fig. 4) also caused inactivation of Akt-mTOR cascade in HUVECs, leading to decreased phosphorylation of Akt-S6K1 (Fig. 6B). Similar to the shRNA experiments, the total protein expression of Akt-S6K1 remained unaltered (Fig. 6B). Conversely, in HUVECs cells overexpressing NDUFS8, “oeNDUFS8” (see Fig. 5), there was an upregulation in Akt-S6K1 phosphorylation (Fig. 6C). These findings collectively suggest that NDUFS8 promotes activation of Akt-mTOR pathway in HUVECs.

Crucially, supplementing ATP effectively mitigated the Akt-mTOR inhibition induced by kdNDUFS8-sh5 in HUVECs (Fig. 6D), implying that mitochondrial dysfunction and ATP depletion might be the primary mechanism of Akt-mTOR inhibition in endothelial cells with silenced NDUFS8. Subsequently, lentivirus carrying the constitutively active (S473D) mutant Akt1 (caAkt1) was introduced to kdNDUFS8-sh5-expressing HUVECs, and stable cells established through puromycin-mediated selection. Figure 6E confirmed the expression of caAkt1 (no Taq), which led to the restoration of Akt-S6K1 phosphorylation in HUVECs expressing kdNDUFS8-sh5 (Fig. 6E). Significantly, the introduction of caAkt1 markedly alleviated the inhibitory effects induced by kdNDUFS8-sh5 on cell proliferation (tested by nuclear EdU staining, Fig. 6F), migration (Fig. 6G), and capillary tube formation (Fig. 6H) in HUVECs.

### Endothelial knockdown of NDUFS8 inhibits retinal angiogenesis in mice

**Fig. 7:**
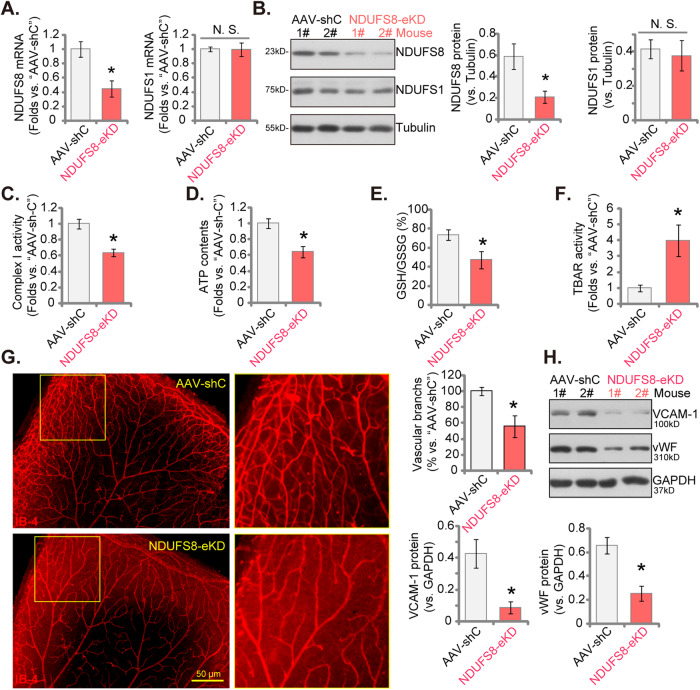
Endothelial knockdown of NDUFS8 inhibits retinal angiogenesis in mice. The adult C57BL/6 mice were intravitreously administered with either murine AAV5-TIE1-NDUFS8 shRNA (“NDUFS8-eKD,” 0.12 μL) or AAV5-TIE1 control scramble shRNA (“AAV-shC”, 0.12 μL). After a duration of twenty-one days, the murine retinal tissues were collected and tests were conducted on the expression levels of various mRNAs and proteins within fresh tissue lysates (**A**, **B**, **H**). The mitochondrial respiratory chain Complex I activity (**C**), cellular ATP levels (**D**), the ratio of reduced to oxidized glutathione (GSH/GSSH ratio) (**E**), and the intensity of thiobarbituric acid reactive substances (TBAR) (**F**) in retinal tissues were also measured. In addition, the retinal vasculatures were measured through retinal isolectin B4 (IB4) staining (**G**). The data are presented as mean ± standard deviation (SD, *n* = 5). *P < 0.05 compared to “AAV-shC” group. “N. S.” represents non-statistically significant disparities (*P* > 0.05). These experiments were repeated five times, yielding consistent results. Scale bar = 100 μm.

To investigate the potential impact of NDUFS8 on in vivo angiogenesis, we conducted experiments on the mouse retinal vasculature, as previously detailed [24]. Adult mice were initially subjected to intravitreal injection of murine AAV5-TIE1-NDUFS8 shRNA, incorporating the sequence of endothelial cell-specific promoter TIE1 [23, 25]. This intervention can effectively lead to the knockdown of NDUFS8 only in endothelial cells, referred to as “NDUFS8-eKD.” As a genetic control treatment, we administered murine AAV5-TIE1-scramble control shRNA (“AAV-shC”) [24] to mouse retina. Twenty-one days post-virus injection, murine retinal tissues were collected, and tissue lysates were analyzed. In NDUFS8-eKD mice, *NDUFS8* mRNA (Fig. 7A) and protein (Fig. 7B) expression was notably reduced, while *NDUFS1* mRNA (Fig. 7A) and protein (Fig. 7B) expression remained unchanged. Furthermore, endothelial knockdown of NDUFS8 resulted in decreased mitochondrial Complex I activity (Fig. 7C) and reduced ATP contents (Fig. 7D) in retinal tissues, supporting the impairment of mitochondrial functions. Moreover, the GSH/GSSG ratio was reduced (Fig. 7E), suggesting heightened oxidative stress in NDUFS8-eKD murine retinal tissues. The increased TBAR intensity further supported lipid peroxidation in the retinal tissues (Fig. 7F).

The examination of retinal vasculature via IB4 staining revealed robust inhibition of angiogenesis in the mouse retina following endothelial knockdown of NDUFS8 (Fig. 7G). NDUFS8-eKD mice exhibited a significantly reduced number of retinal vascular branches and branch points (Fig. 7G). In addition, two endothelial marker proteins, von Willebrand factor (vWF) and VCAM-1 [24], were downregulated in retinal tissues following NDUFS8-eKD (Fig. 7H). Therefore, endothelial knockdown of NDUFS8 inhibited retinal angiogenesis in mice.

### NDUFS8 overexpression in proliferative membrane tissues of proliferative diabetic retinopathy (PDR) patients

**Fig. 8:**
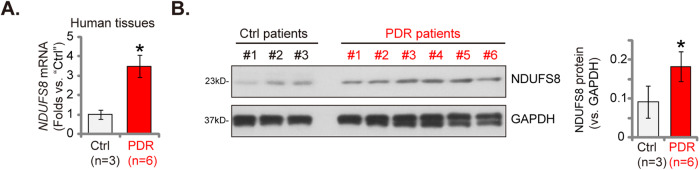
NDUFS8 overexpression in proliferative membrane tissues of proliferative diabetic retinopathy (PDR) patients. The human tissues listed underwent homogenization and were subsequently assessed the mRNA and protein expression of NDUFS8 (**A**, **B**, *n* = 3/6). The data are presented as mean ± standard deviation (SD). *P < 0.05 compared to “Ctrl” tissues.

Proliferative diabetic retinopathy (PDR) is characterized by the growth of abnormal blood vessels in the retina due to angiogenesis, a process triggered by chronic high blood sugar levels in individuals with diabetes [48–50]. We therefore examined the expression of NDUFS8 in the proliferative retinal tissues of PDR patients. Our investigation involved the analysis of previously documented human tissues [25, 28, 34]. The retinal proliferative membrane tissues from six distinct PDR patients were collected, in addition to retinal tissues from three control patients who underwent traumatic retinectomy and were matched for age (“Ctrl”) [25, 28]. The data presented in Fig. 8A, B revealed a significant increase in *NDUFS8* mRNA and protein expression within the proliferative retinal membrane tissues of individuals with PDR. These findings provide further evidence supporting the potential involvement of NDUFS8 in the development of pathological retinal angiogenesis.

## Discussion

Angiogenesis is the process of forming new blood vessels. Endothelial cells, which line existing blood vessels, play a vital role in this process [6–8]. They sprout, proliferate, migrate, and organize to create new blood vessels [6–8]. Mitochondria, the cellular powerhouses responsible for energy production, provide the essential ATP required for endothelial cell proliferation, migration, sprouting, capillary tube formation, and vessel remodeling during angiogenesis [9, 10]. In addition, mitochondria are involved in redox signaling pathways critical for regulating angiogenic processes [9, 10]. Dysfunctional mitochondria can disrupt angiogenesis, and contributing to the pathogenesis of various cardiovascular and metabolic disorders [9, 10].

Early research has illuminated the critical role of specific mitochondrial components in endothelial activation and angiogenesis. Wang et al., reported that endothelial knockdown of mitochondrial outer-membrane protein FUNDC1 (FUN14 domain-containing protein 1) resulted in reduced vascular endothelial growth factor receptor 2 (VEGFR2) expression, and hindered tube formation, spheroid-sprouting in vitro and angiogenesis in vivo [51]. In endothelial progenitor cells, blocking pyruvate kinase muscle isoenzyme 2 (PKM2) through C3k-mediated mechanisms led to the downregulation of angiogenesis-associated genes and inhibited tube formation [52]. In addition, mitochondrial dysfunction and oxidative stress were observed in C3k-stimulated endothelial progenitor cells [52]. Our recent study has further contributed to this understanding by demonstrating that genetic depletion or pharmacological inhibition of TIMM4 (translocase of inner mitochondrial membrane 44), an inner mitochondrial membrane protein [53], impaired the mitochondrial functions and impeded angiogenesis in vitro and in vivo [24].

**Fig. 9:**
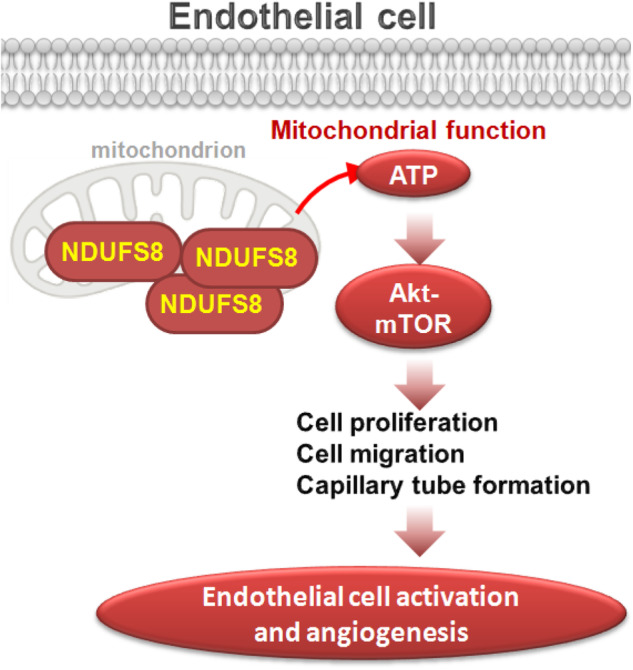
The proposed signaling carton of this study. Enhancing mitochondrial function and ATP production via NDUFS8 is vital for activating the Akt-mTOR pathway, thereby promoting endothelial cell activation and facilitating angiogenesis.

NDUFS8 is a critical subunit of Complex I, involved in the electron transport chain within the mitochondria, where it helps to transfer electrons and facilitates the generation of ATP. In the present study, we investigated the role of NDUFS8 in mitochondrial functions of endothelial cells. NDUFS8 silencing (by targeted shRNAs) or KO (through CRISPR/Cas9 method) impaired mitochondrial functions within various endothelial cells, causing a reduction in mitochondrial Complex I activity, decreased ATP production, mitochondrial depolarization, increased oxidative stress and ROS production, and intensified lipid oxidation. Contrarily, ectopic overexpression of NDUFS8, using a lentiviral construct, augmented mitochondrial Complex I activity and increased ATP contents in HUVECs and other endothelial cells. Importantly, mitochondrial function impairment, ATP reduction, oxidative stress, and enhanced lipid oxidation were detected in mouse retinal tissues with endothelial knockdown of NDUFS8. All of these evidence indicate that NDUFS8 is crucial for maintaining mitochondrial functions and overall cellular energy production within endothelial cells (see proposed signaling carton in Fig. 9).

We have presented compelling data demonstrating the pivotal role of the mitochondrial protein NDUFS8 in regulating endothelial cell activation and the angiogenic process. Specifically, the silencing or knockout of NDUFS8 impeded cell proliferation, migration, invasion, and capillary tube formation in various endothelial cell types, including HUVECs, hRMEC, hDEC, and hCMEC. NDUFS8 depletion led to an increase in apoptosis among endothelial cells. Significantly, the exogenous addition of ATP or the antioxidant NAC effectively mitigated the anti-angiogenic effects by NDUFS8 shRNA in cultured endothelial cells. Ectopic overexpression of NDUFS8, achieved through a lentiviral construct, demonstrated a pro-angiogenic impact by enhancing cell proliferation, migration, and capillary tube formation in HUVECs and other endothelial cell types. In vivo, the intravitreous administration of an endothelial-specific NDUFS8 shRNA AAV inhibited retinal angiogenesis (Fig. 9).

Our results suggest that NDUFS8 plays a crucial role in promoting Akt-mTOR pathway activation in endothelial cells, the key cascade for endothelial cell activation and angiogenesis [45–47]. NDUFS8 depletion led to inhibition of Akt-mTOR activation, which was restored by exogenous ATP supplementation. Conversely, NDUFS8 overexpression boosted Akt-mTOR activation. Furthermore, the inhibitory effects of NDUFS8 knockdown on cell proliferation, migration, and capillary tube formation were rescued by via caAkt1. This suggests that the promotion of mitochondrial function and ATP production by NDUFS8 are crucial for activating the Akt-mTOR pathway, consequently facilitating endothelial cell activation and angiogenesis (Fig. 9).

PDR stands as a severe and vision-endangering complication intricately intertwined with angiogenesis [50, 54–56]. In PDR, the chronic state of hyperglycemia and associated vascular damage stemming from diabetes mellitus culminates in the formation of anomalous and frail retinal blood vessels [50, 54–56]. These neo-vascular structures are a direct consequence of uncontrolled angiogenesis, primarily driven by the presence of hypoxia, or oxygen deficiency, and the concomitant release of various angiogenic growth factors, most notably VEGF [50, 54–56]. The excessive angiogenesis observed in PDR subsequently leads to the development of delicate, permeable vessels, often resulting in retinal hemorrhage, fibrosis, and ultimately, severe vision impairment [50, 54–56]. In the present study, we showed that NDUFS8 expression, at both mRNA and protein levels, is increased in proliferative membrane tissues of PDR patients, suggesting that the mitochondrial protein could be a promising therapeutic target of PDR and possible other disease characterized by pathological angiogenesis.

### Supplementary information

SUPPLEMENTAL Figures

## Data Availability

All data are available upon reasonable request.
