# Supplementary material for: The requirement of the mitochondrial protein NDUFS8 for angiogenesis
Source: Cell Death Dis. 2024 Apr 9;15(4):253. doi: 10.1038/s41419-024-06636-3 (PMC11004167; doi:10.1038/s41419-024-06636-3)
Supplement: Supplementary file 1 — SUPPLEMENTAL Figures [file 41419_2024_6636_MOESM1_ESM.pdf]

Figure S1.

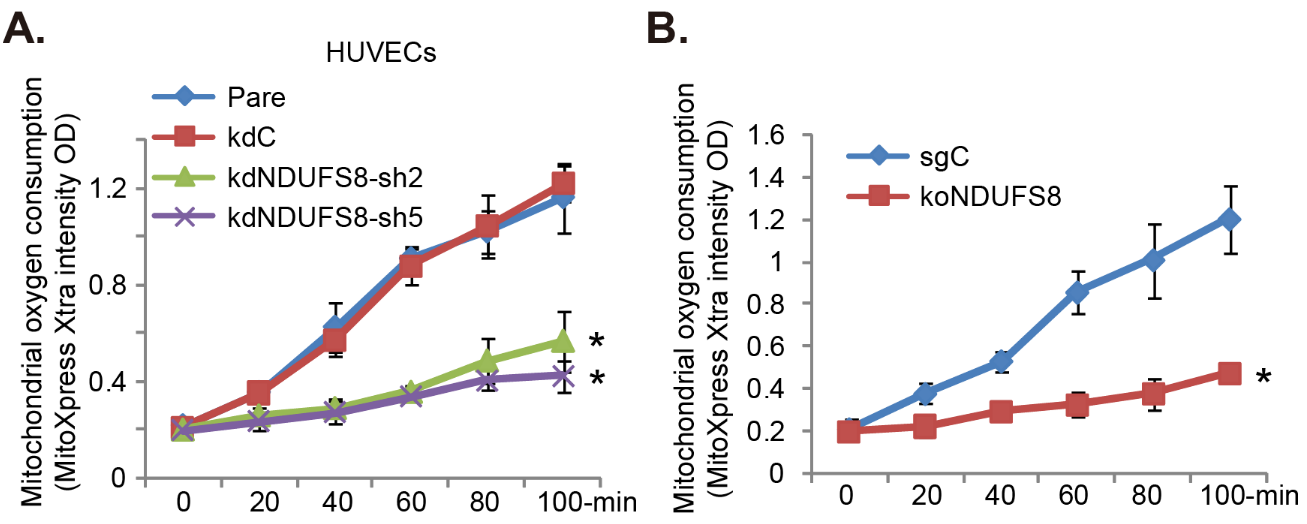

Figure S1. HUVECs with NDUFS8 shRNA ("kdNDUFS8-sh2" and "kdNDUFS8-sh5"), the scramble non-sense shRNA ("kdC") (**A**), the Cas9-expressing construct plus the CRISPR/Cas9-NDUFS8-KO construct ("koNDUFS8") or the control construct ("sgC") (**B**) were cultivated for indicated time periods, the mitochondrial oxygen consumption was tested via quantifying MitoXpress Xtra fluorescence intensity. The data are presented as mean ± standard deviation (SD, n = 5). * ***P*** < 0.05 compared to "kdC"/"sgC" cells. These experiments were repeated five times, yielding consistent results.

Figure S2

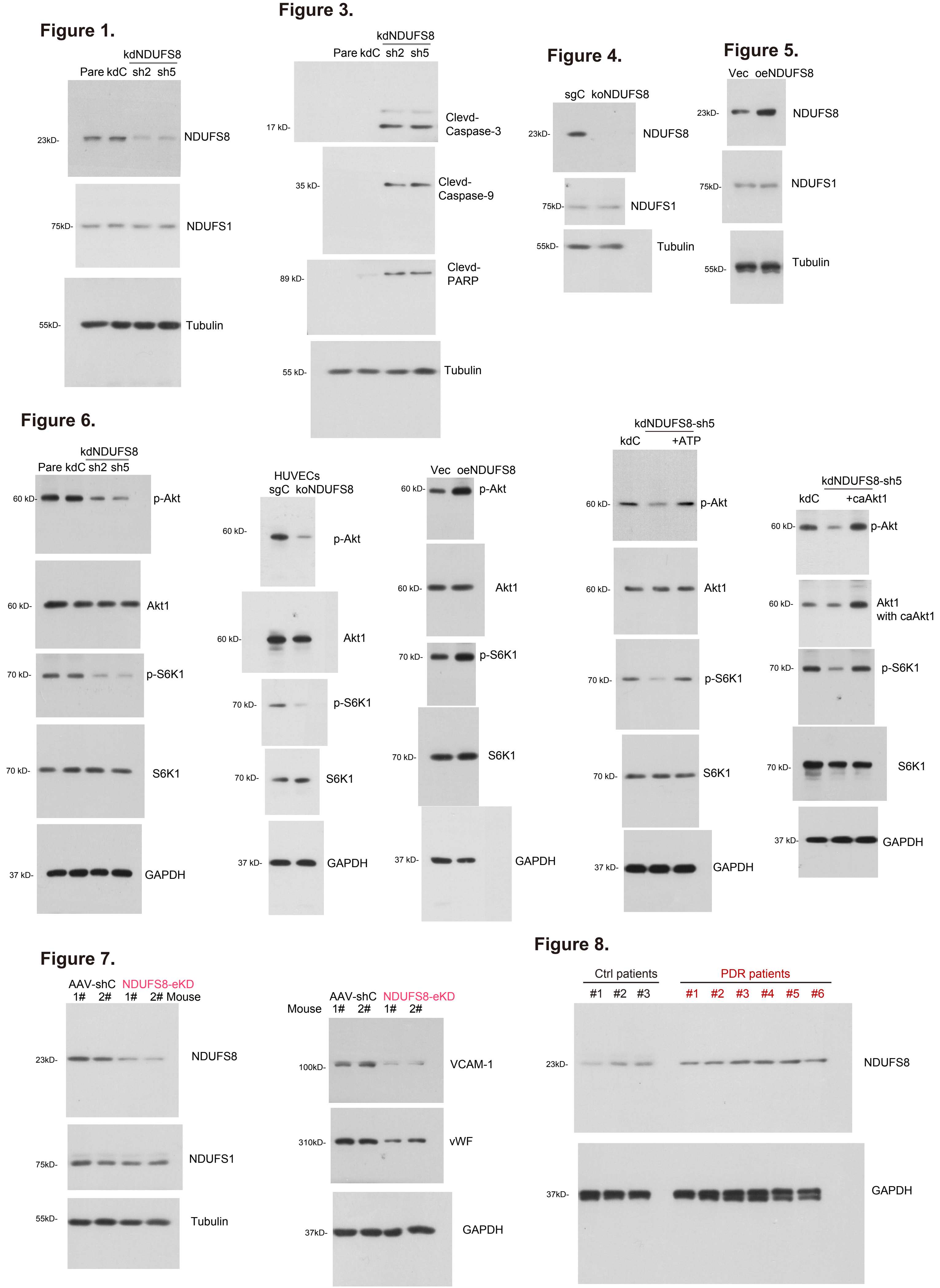

Figure S2. The uncropped blotting images.
